# Characterization of oral microbiome alterations in thalassemia patients using 16S rRNA gene sequencing

**DOI:** 10.3389/fmicb.2026.1853312

**Published:** 2026-08-17

**Authors:** Mashail A. Alghamdi, Shoog A. Almalki, Ahmed Bahieldin, Osman Radhwi, Irfan A. Rather

**Affiliations:** 1 Department of Biological Sciences, Faculty of Science, King Abdulaziz University, Jeddah, Saudi Arabia; 2 Department of Genetics, Faculty of Agriculture, Ain Shams University, Cairo, Egypt; 3 Department of Hematology, Faculty of Medicine, King Abdulaziz University, Jeddah, Saudi Arabia; 4 Centre of Excellence in Bionanoscience Research, King Abdulaziz University, Jeddah, Saudi Arabia

**Keywords:** 16S rRNA, oral microbiome, *Porphyromonas gingivalis*, *Prevotella*, thalassemia

## Abstract

**Background:**

This study investigates the composition of the oral microbiota in thalassemia patients and compares it with healthy individuals from Saudi Arabia. Thalassemia is an inherited blood disorder characterized by reduced or absent production of globin chains, resulting in hypochromic microcytic anemia and multiple complications, such as iron overload resulting from frequent blood transfusions.

**Methods:**

Using 16S rRNA gene sequencing, 28 saliva samples (14 thalassemia patients and 14 healthy controls) were analyzed to assess changes in the richness and diversity of the oral microbiota.

**Results:**

Taxonomic analysis demonstrated nominal differences between the two groups, with notable variations in the abundance of several bacterial species. All taxa showing differential abundance belonged to two phyla-Firmicutes and Bacteroidota; including taxa such as *Prevotella intermedia, Eubacterium sulci*, and *Porphyromonas gingivalis*. Although several taxa showed nominal differences based on raw p-values, none remained statistically significant after Benjamini-Hochberg false discovery rate (FDR) correction. Thalassemia patients exhibited a nominal reduction in the relative abundance of bacteria such as *Prevotella*.

**Conclusion:**

Overall, this study suggests possible alterations in the oral microbiome associated with thalassemia; however, these findings require validation in larger cohorts with comprehensive clinical and oral health data.

## 1 Introduction

Thalassemia is a hereditary hypochromic microcytic anemia caused by reduced or absent synthesis of globin chains, which results in defective hemoglobin production (Cao and Galanello, 2010). It is an autosomal recessive disorder historically prevalent in the Mediterranean, the Middle East, Southeast Asia, and the Indian subcontinent. However, global migration has expanded its distribution to North America, South America, Australia, and Northern Europe (Yousuf et al., 2022). Hemoglobin is composed of heme and globin components and is responsible for oxygen transport from the lungs to body tissues. Adult hemoglobin consists of two α and two β globin chains, although the exact proportions may vary depending on age, genetic background, and underlying health conditions (Galanello and Cao, 2011).

Thalassemia is broadly classified into α-thalassemia and β-thalassemia. Globally, an estimated 300,000–400,000 infants are born annually with severe hereditary hemoglobin disorders, while around 80 million individuals are estimated to be carriers of β-thalassemia (Yousuf et al., 2022). In Saudi Arabia, the α-thalassemia is highly prevalent, although its most severe form, homozygous α-thalassemia (hydrops fetalis syndrome), has not been reported. β-thalassemia is also common, particularly in the Eastern Province and along the Red Sea coast, including Jubail, Qatif, Dammam, and Hofuf (Al-Awamy, 2000).

The oral microbiome consists of diverse microbial communities that exist in a dynamic equilibrium important for balancing oral and systemic health (Sandrini et al., 2015; Singh et al., 2017; Tagg et al., 2023). Disruption of this balance, known as oral dysbiosis, has been associated with inflammatory, immune and metabolic disorders (Carding et al., 2015; Khalil et al., 2022; Tagg et al., 2023). The oral cavity harbors one of the most diverse microbial ecosystems in the human body, with anaerobic bacteria representing a major component of this community (Könönen and Gursoy, 2022; Könönen et al., 2022). Advances in next-generation sequencing (NGS) technologies have greatly improved the characterization of these complex microbial communities and enabled comprehensive microbiome profiling (Aggarwal et al., 2023).

Several clinical features of thalassemia, including chronic anemia, repeated blood transfusions, iron overload, immune dysregulation, and associated oral manifestations, may influence the oral microbial ecosystem. Consequently, alterations in oral microbial composition could contribute to changes in oral health in affected individuals. However, despite increasing interest in host–microbiome interactions, studies investigating the oral microbiome in patients with thalassemia remain limited.

The present study aimed to characterize the oral microbiome of thalassemia patients using 16S rRNA gene sequencing and compare microbial profiles with those of healthy individuals from Jeddah, Saudi Arabia.

## 2 Materials and methods

### 2.1 Sample collection and study design

A case-control study involving volunteers from the Makkah region was conducted between November and December 2023. Participants were recruited from King Abdulaziz University Hospital (KAUH), Jeddah, Saudi Arabia. Written informed consent was obtained from all participants, and the study was approved by the Unit of Biomedical Ethics Research Committee, Faculty of Medicine, King Abdulaziz University, Jeddah, Saudi Arabia. Ethical approval was granted on Thursday, November 2, 2023, under reference number 533–23.

Saliva samples were collected from participants who met the study eligibility criteria. The study included 14 clinically diagnosed thalassemia patients and 14 healthy volunteers (control group). Both groups were equally distributed by sex (7 male and 7 female), with participant ages ranging from 14 to 44 years.

Individuals with chronic diseases, current or recent antibiotics use, smoking, pregnancy or consumption of probiotics or prebiotics within the 3 months prior to sample collection were excluded.

For sample collection, participants were instructed to refrain from both brushing and the use of mouthwash before saliva collection. In addition, participants were instructed not to consume food or beverages for at least 2 h prior to sample collection.

### 2.2 DNA extraction

Each participant was provided with a saliva DNA microbiome collection tube containing a stabilizing buffer. Participants were instructed to spit into the funnel until the liquid reached the 2 mL mark while avoiding bubble formation. Following this, the tubes were then shaken by each participant or, when applicable, by a parent or guardian to ensure thorough mixing of the samples with the stabilizing solution. Samples were transported to the King Fahad Center and stored at -80°C until analysis.

Microbial DNA extraction was carried out using the QIAamp DNA Mini Kit (50) in accordance with the manufacturer’s instructions. Briefly, 400 μL of saliva was transferred into a 1.5 mL microcentrifuge tube, followed by the addition of 360 μL of Buffer ATL and 40 μL of Proteinase K. The mixture was vortexed and incubated at 56°C for 30 min. Subsequently, 200 μL of Buffer AL was added and mixed for 15 s. The samples were then incubated at 70°C for 10 min, followed by the addition of 200 μL of ethanol (96–100%) and vortexing for another 15 s.

The mixture was transferred to a QIAamp Mini spin column in a 2 mL collection tube and centrifuged at 6,000 × g for 1 min. The filtrate was discarded, and 500 μL of Buffer AW1 was added, followed by centrifugation under the same conditions. After that, 500 μL of Buffer AW2 was added, and the column was centrifuged at 20,000 × g for 3 min. The filtrate was discarded, and the spin column was transferred to a clean 1.5 mL microcentrifuge tube. DNA was eluted by adding 30 μL of Buffer AE or distilled water, followed by incubation at room temperature for 30 min, and centrifugation at 6,000 × g for 10 s. The elution step was repeated using 20 μL of Buffer AE to increase DNA yield.

### 2.3 DNA purification and concentration measurement

The quantity and purity of DNA were assessed using NanoDrop 2000 spectrophotometer (Thermo Scientific). DNA concentration and purity were evaluated using the A260/280 ratio for protein contamination and the A260/230 ratio for salt and organic compounds.

### 2.4 Gel electrophoresis

Agarose gel electrophoresis was performed for the separation of DNA fragments based on size. The gel was prepared following the methodology described elsewhere (Lee et al., 2012). Briefly, a 1% agarose gel was prepared by dissolving 1 g of agarose powder in 100 mL of distilled water in an Erlenmeyer flask. The mixture was thoroughly mixed until the agarose was completely dissolved. The solution was then heated in a microwave at 30-s intervals for a total of 2 min to ensure complete dissolution.

The gel was prepared using TAE running buffer, and ethidium bromide (EtBr) was added as a fluorescent agent at a final concentration of 0.5 μg/mL. After cooling, the agarose solution was poured into the gel mold and allowed to solidify before use.

### 2.5 Bioinformatics and statistical analysis

Raw paired-end sequencing reads were processed using a standardized bioinformatics workflow. Adapter sequences and low-quality reads were removed using fastp (v0.23.1) to obtain high-quality clean reads. Paired-end reads were merged using FLASH (v1.2.11), and chimeric sequences were identified using the UCHIME algorithm implemented in VSEARCH (v2.16.0) against the SILVA reference database (version 138.2) and removed.

High-quality reads were subsequently processed using the DADA2 pipeline within QIIME2 (version 2022.2) to generate Amplicon Sequence Variants (ASVs). Taxonomic assignment was performed using the SILVA database (version 138.2), while sequences that remained unclassified were further annotated using the Micro_NT database employing BLAST-based alignment and the least common ancestor (LCA) algorithm.

A diversity was evaluated using the Observed Features, Chao1, Shannon, and Simpson indices. β diversity was assessed using weighted and unweighted UniFrac distance matrices and visualized by Principal Coordinate Analysis (PCoA). Analysis of similarity (ANOSIM) was performed to evaluate differences in microbial community structure between study groups.

Differential abundance analyses were performed to compare bacterial taxa between groups. To account for multiple comparisons, raw *P*-values were adjusted using the Benjamini–Hochberg false discovery rate (FDR) method, and adjusted *q*-values < 0.05 were considered statistically significant.

### 2.6 Polymerase chain reaction

PCR amplification was performed using Phusion^®^ High-Fidelity PCR Master Mix (New England Biolabs) with barcoded universal primers targeting the V4 region of the bacterial 16S rRNA gene (primers 515F: 5′-GTGCCAGCMGCCGCGGTAA-3′/806R: 5′-GGACTACHVGGGTWTCTAAT-3′). Following amplification, PCR products were purified using magnetic bead purification (e.g., AMPure XP). DNA concentrations were normalized, and samples were pooled in equidensity ratios.

Library quality and insert size were assessed using the Agilent 2100 Bioanalyzer, and library concentrations were quantified using a Qubit fluorometer and real-time PCR. Final libraries were sequenced on an Illumina platform, generating ∼250 bp paired-end reads.

## 3 Results

### 3.1 Participant characteristics

A total of 28 participants were included in the study, comprising 14 clinically diagnosed thalassemia patients and 14 healthy controls. Both groups included seven males and seven females. The mean age was 26.6 ± 9.5 years in the thalassemia group and 27.8 ± 9.7 years in the healthy control group. The baseline demographic characteristics of the study participants are summarized in Table 1.

**TABLE 1:** Baseline characteristics of the study participants.

Characteristic	Thalassemia (*n* = 14)	Healthy controls (*n* = 14)
Age (years), mean ± SD	26.6 ± 9.5	27.8 ± 9.7
Age range (years)	14–44	14–42
Male, n (%)	7 (50%)	7 (50%)
Female, n (%)	7 (50%)	7 (50%)

### 3.2 DNA yield and quality assessment

DNA was successfully extracted from all 28 saliva samples, with concentrations ranging from 13.2 to 113.1 ng/μL. The highest DNA yield was obtained from a healthy control sample (113.1 ng/μL), whereas the lowest concentration was observed in a thalassemia sample (13.2 ng/μL). Furthermore, DNA integrity was assessed by agarose gel electrophoresis, and clear DNA bands were observed for all samples, indicating that the extracted DNA was of sufficient quality for downstream sequencing analysis.

### 3.3 Statistics of the oral microbiota composition

Illumina MiSeq sequencing was performed on 28 saliva samples collected from thalassemia patients and healthy controls using 16S rRNA gene amplicon sequencing. The sequence read lengths ranged from 420.77 to 426.49 base pairs (bp). The average numbers of raw tags, clean tags, and effective tags were 208745.29, 205196.21, and 181430.93, respectively. The mean Q20 and Q30 scores were 98.50 and 94.94%, respectively, indicating sequencing error rates of less than 1 and 0.1%. These results demonstrate that the sequencing data were of sufficient quality for subsequent microbiome analyses.

### 3.4 Annotation at phylum-level

**FIGURE 1:**
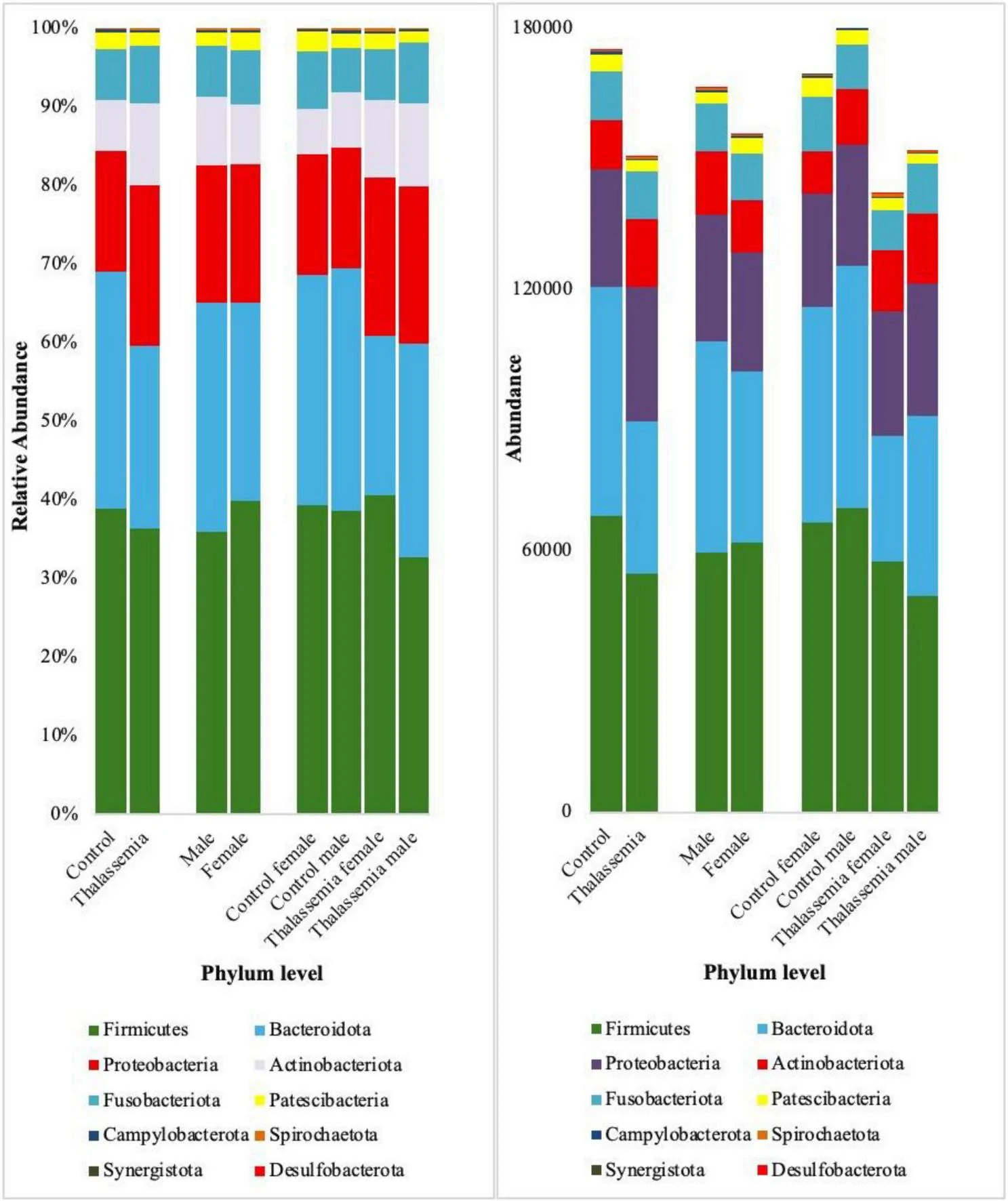
The distribution of oral microbial taxonomic composition at the phylum level, based on the abundance and relative abundance of different phyla in the thalassemia patients and healthy control groups.

The 10 most abundant bacterial phyla identified in thalassemia patients and healthy controls are presented in Figure 1. Firmicutes showed the highest abundance in both groups, followed by Bacteroidota, Proteobacteria, Actinobacteriota, Fusobacteriota, Patescibacteria, Campylobacterota, Spirochaetota, Synergistota, and Desulfobacterota.

A lower relative abundance of Firmicutes and Bacteroidota was observed in thalassemia patients compared with healthy controls, whereas Proteobacteria and Actinobacteriota exhibited relatively higher abundance. The relative abundance of Firmicutes, Bacteroidota, Proteobacteria, and Actinobacteriota in the thalassemia patients were 40, 26, 23, and 11%, respectively, whereas in healthy controls, the corresponding values were 43, 33, 17, and 7%, respectively.

In the sex-based comparison, a slight decrease in the relative abundance of Firmicutes and a slight increase in Bacteroidota were observed in males compared with females. Among the subgroups, Firmicutes showed higher relative abundance in control males but lower abundance in male thalassemia patients, whereas Bacteroidota showed higher abundance in control males and lower abundance in female thalassemia patients. Nonetheless, these phylum level differences were not statistically significant across the study groups.

#### 3.4.1 Annotation at genus-level

**FIGURE 2:**
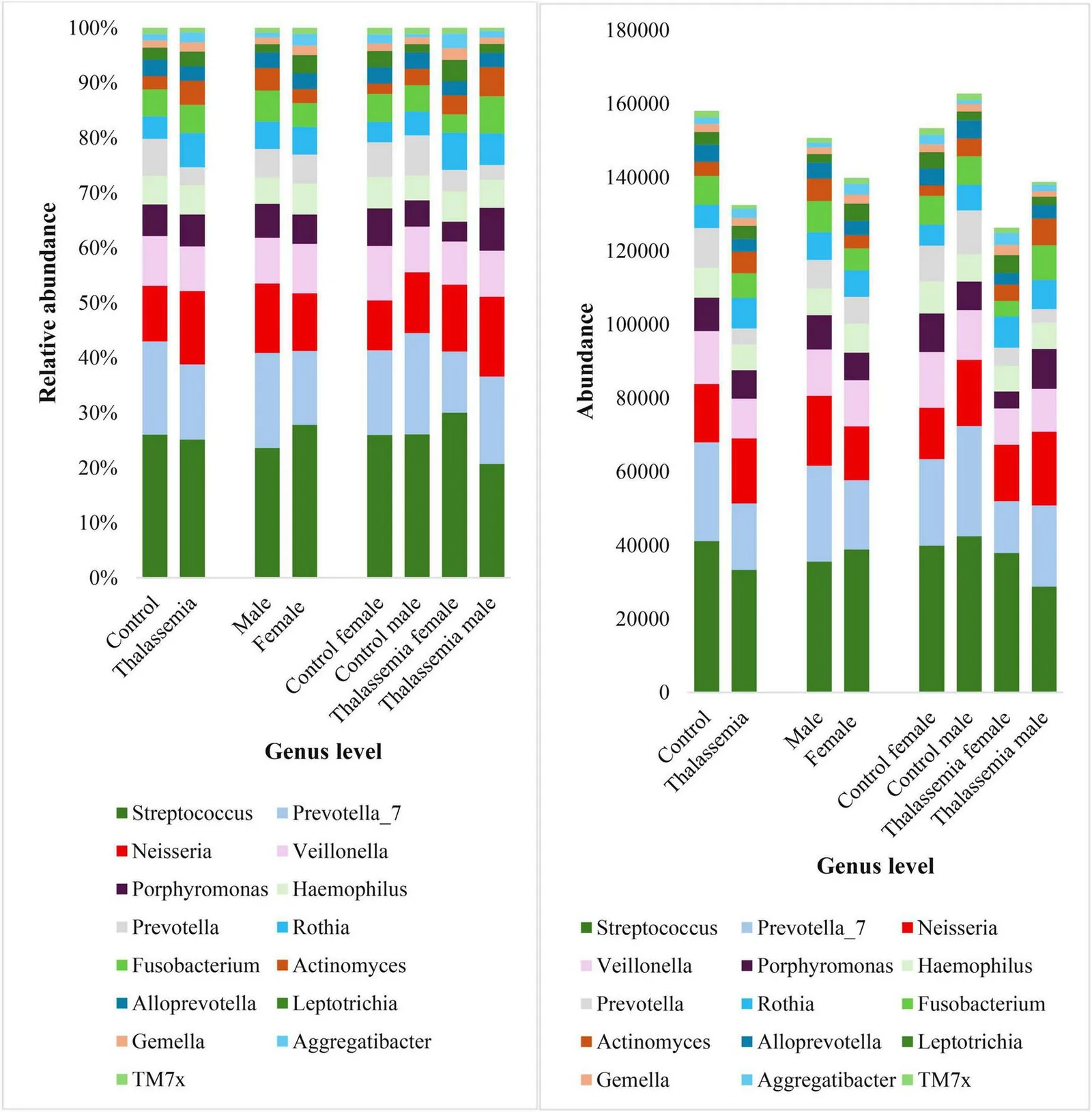
The distribution of oral microbial taxonomic composition at the genus level, based on the abundance and relative abundance in thalassemia patients and healthy control groups.

Figure 2 shows the top 15 most abundant genera identified in thalassemia patients and healthy controls. *Streptococcus* was the most abundant genus in both groups, followed by *Prevotella_7 (*a SILVA-specific taxonomic annotation within the Prevotella genus*)*, *Neisseria*, *Veillonella*, *Porphyromonas*, *Haemophilus*, *Prevotella*, *Rothia*, *Fusobacterium*, *Actinomyces*, *Alloprevotella*, *Leptotrichia*, *Gemella*, *Aggregatibacter*, and *TM7x*.

Overall, differences in the relative abundance of several genera, including *Streptococcus*, *Prevotella_7*, *Neisseria*, and *Prevotella*, were observed between thalassemia patients and healthy controls. In thalassemia samples, *Streptococcus* and *Prevotella_7* showed lower relative abundance, whereas *Neisseria* showed higher relative abundance compared with healthy controls. In addition, the genus *Prevotella*, which showed relatively low overall abundance, was more abundant in the control group than in thalassemia patients based on raw *P*-value (*P* = 0.001) (Supplementary Figure S1). Of note, this association did not remain statistically significant after Benjamini-Hochberg FDR correction.

Exploratory differences in the relative abundance of *Streptococcus*, *Prevotella_7* and *Neisseria* were also observed between male and female subgroups. *Actinomyces* showed higher relative abundance in thalassemia patients than in healthy controls, and was more abundant in males than females, particularly among male thalassemia patients. No statistically significant genus-level differences were observed among the sex-based subgroups. Also, no major differences were observed among the remaining detected genera.

The genus level heat map illustrates the relative abundance patterns of the 35 most abundant genera across the thalassemia and control groups, with *Streptococcus*, *Prevotella_7, Neisseria*, and *Prevotella*, among the most dominant genera.

#### 3.4.2 Annotation at species-level

**FIGURE 3:**
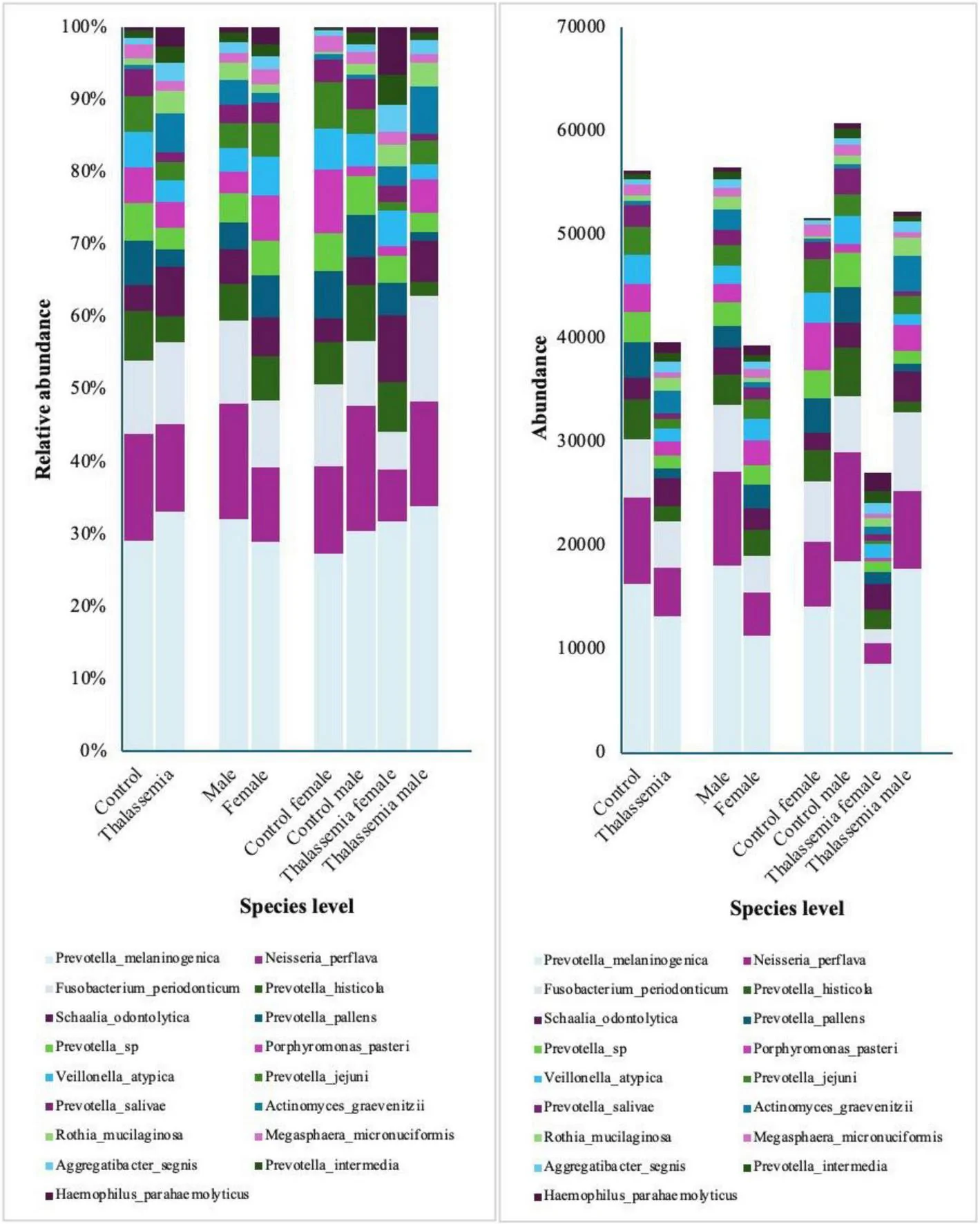
The distribution of oral microbial taxonomic composition at the species level, based on the abundance and relative abundance in thalassemia patients and healthy controls.

As shown in Figure 3, the 17 most abundant species identified across both study groups included *Prevotella_melaninogenica*, *Neisseria_perflava*, *Fusobacterium_periodonticum*, *Prevotella_ histicola*, *Schaalia_odontolytica*, *Prevotella_pallens*, *Prevotella_sp*, *Porphyromonas_pasteri*, *Veillonella_atypica*, *Prevotella_jejuni*, *Prevotella_salivae*, *Actinomyces_graevenitzii*, *Rothia_mucilaginosa*, *Megasphaera_micronuciformis*, *Aggregatibacter_segnis*, *Prevotella_ intermedia*, and *Haemophilus_parahaemolyticus*.

Overall, a higher relative abundance of several species was observed in healthy controls than in thalassemia patients. Thalassemia samples exhibited lower abundance of *Prevotella_melaninogenica*, *Neisseria_perflava*, *Fusobacterium_periodonticum*, *Prevotella_histicola*, *Prevotella_ pallens*, *Prevotella_sp*, *Porphyromonas_pasteri*, *Veillonella_atypica*, *Prevotella_jejuni*, and *Prevotella_salivae* compared to controls. Moreover, *Schaalia_odontolytica*, *Actinomyces_graevenitzii*, *Rothia_mucilaginosa*, *Megasphaera_micronuciformis*, *Aggregatibacter_segnis*, *Prevotella_intermedia*, and *Haemophilus_parahaemolyticus* showed relatively higher abundance in thalassemia patients.

Differences in species-level abundance were also observed for *Prevotella pallens*, *Prevotella* sp., and *Eubacterium sulci*, which showed lower abundance in thalassemia patients than in healthy controls based on raw *P*-values (*P* = 0.018, *P* = 0.016, *P* = 0.034, respectively). whereas *Porphyromonas gingivalis* showed higher abundance in thalassemia patients (*P* = 0.026) (Supplementary Figure S2). Nevertheless, none of these associations remained statistically significant after Benjamini-Hochberg FDR correction.

Likewise, several species showed variation between male and female subgroups. No statistically significant species-level differences were observed among the sex-based subgroups. A summary of the taxa-level differential abundance analysis before and after Benjamini–Hochberg FDR correction is shown in Table 2. Several taxa demonstrated nominal significance based on the raw *P*-values. However, none remained statistically significant after Benjamini–Hochberg FDR correction.

**TABLE 2:** Taxa level differential abundance analysis before and after FDR correction.

Taxonomic level	Taxa with raw *P* < 0.05	Taxa significant after FDR correction (*q* < 0.05)
Family	1	0
Genus	3	0
Species	7	0

### 3.5 Genus phylogenetic analysis

**FIGURE 4:**
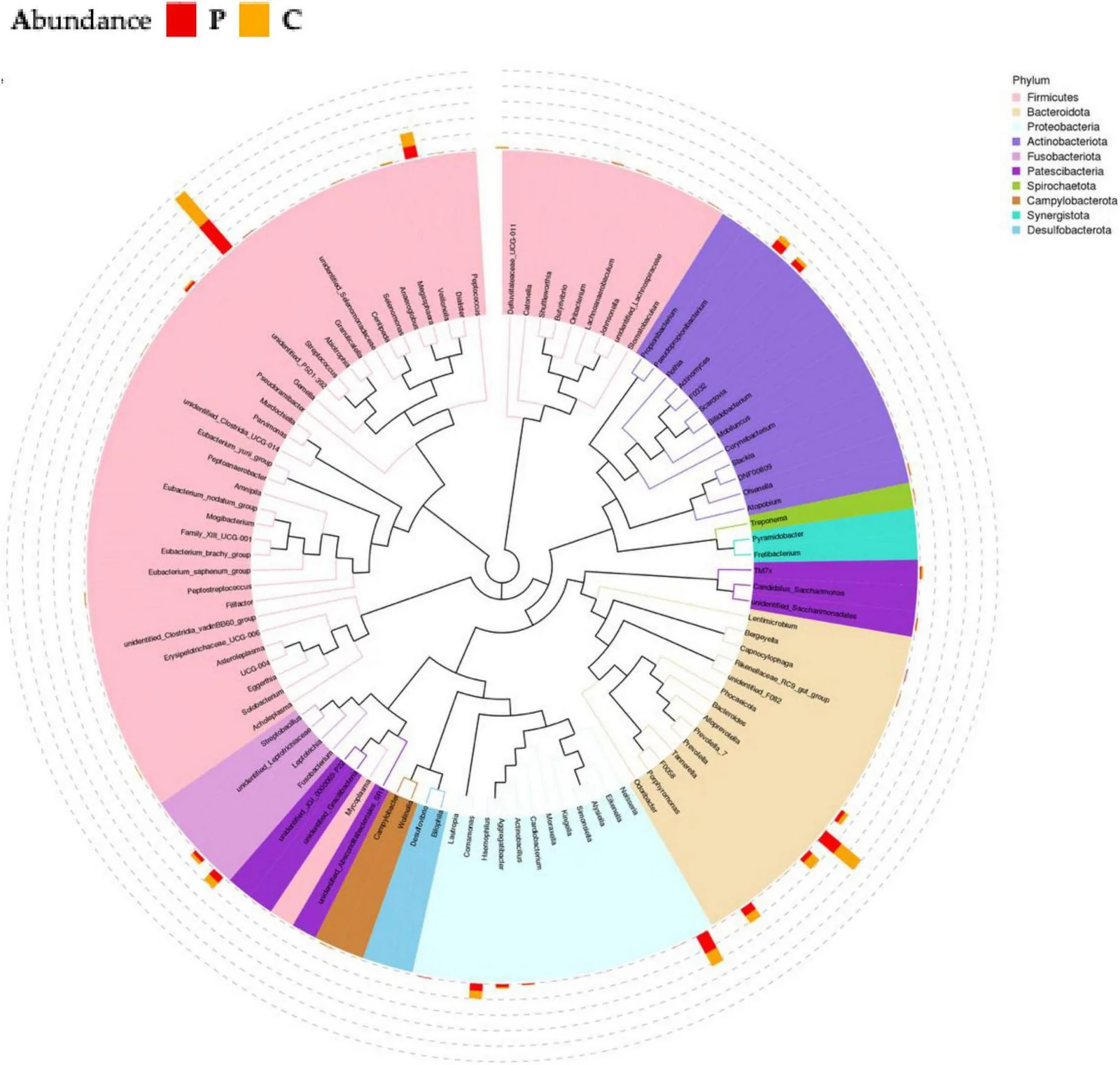
Phylogenetic tree of the 100 most abundant genera identified in microbiome samples collected from thalassemia patients (P) and health controls (C).

Figure 4 presents the phylogenetic relationships among the 100 most abundant genera identified across all samples. The phylogenetic distribution was consistent with the taxonomic composition and relative abundance patterns observed in the thalassemia and healthy control groups.

### 3.6 Venn and flower diagrams

The Venn diagram showed the presence of 1,101 shared amplicon sequence variants (ASVs) between the thalassemia and healthy control groups. A total of 1,483 unique ASVs were identified in the thalassemia group, whereas 1,255 unique ASVs were identified in the control group (Supplementary Figure S3). The shared ASVs represent microbial taxa detected in both study groups, whereas the unique ASVs were detected exclusively in one group. Most of the unique ASVs were of low abundance or present in only a few individuals and therefore contributed minimally to the overall microbial composition of the group.

**FIGURE 5:**
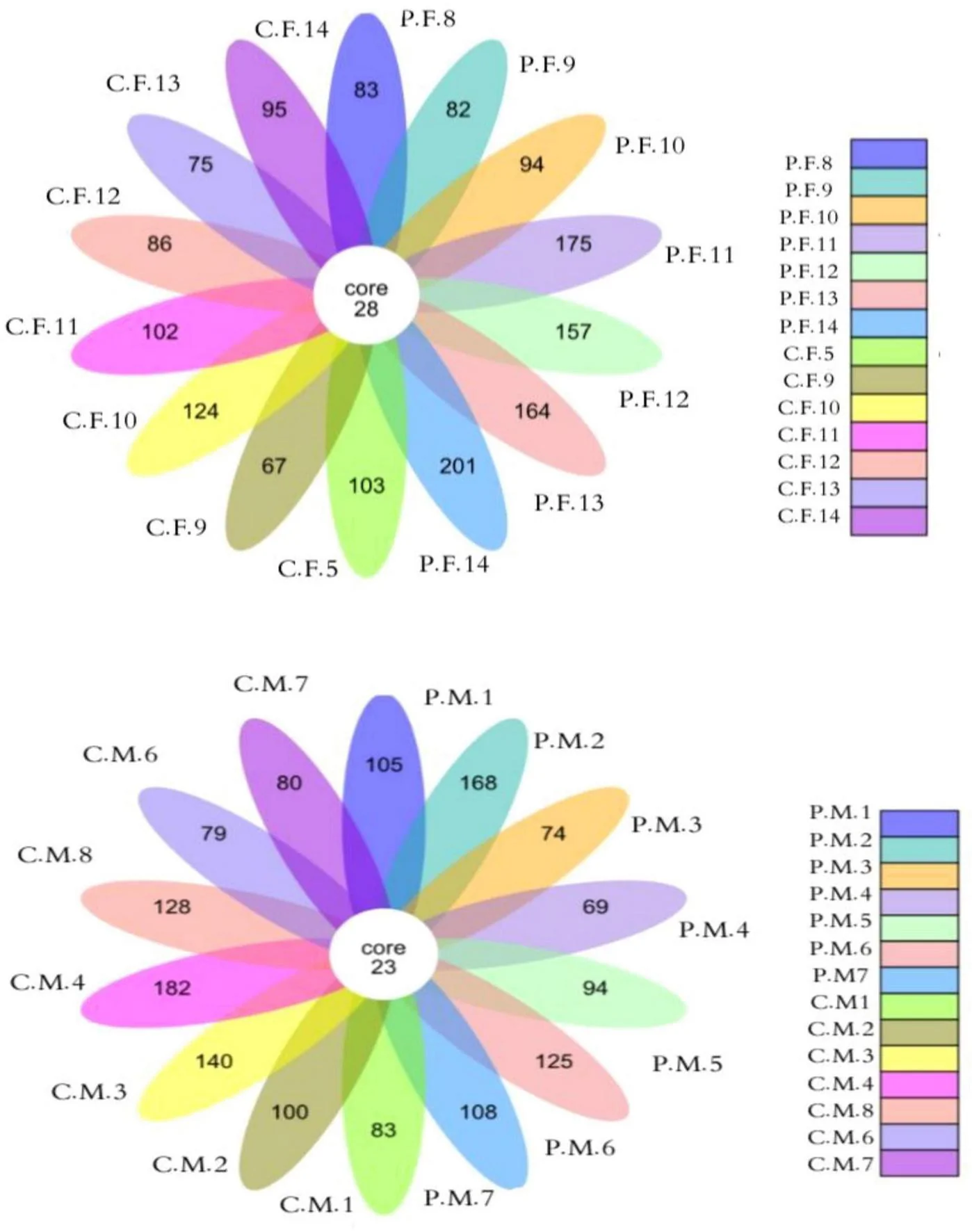
Flower diagram depicting the ASVs in oral microbiomes among male thalassemia patients (P.M) and healthy control males (C.M), as well as female thalassemia patients (P.F) and healthy control females (C.F) for all individuals. The diagram indicates the unique and group-shared ASVs across the 14 groups for both males and females.

The flower diagram analysis indicated that the number of shared ASVs between healthy males and thalassemia patients was 23, whereas 28 shared ASVs were identified between healthy females and thalassemia patients (Figure 5). A total number of 16 ASVs were shared across all study groups, including ASV0, ASV1, ASV2, ASV3, ASV13, ASV15, ASV21, ASV23, ASV32, ASV49, ASV52, ASV60, ASV73, ASV80, ASV96, and ASV119.

### 3.7 Diversity analysis

Diversity analysis was performed to assess the richness, diversity, and composition of the oral microbial communities in thalassemia patients and healthy controls. Rarefaction and species accumulation analyses were used to evaluate sequencing depth and sampling adequacy, while α-diversity was used to assess within-sample microbial diversity and β-diversity was used to compare microbial community composition between the study groups.

#### 3.7.1 Data rarefaction

Rarefaction analysis was performed to evaluate whether the sequencing depth was sufficient to capture the majority of microbial diversity across samples. As shown in Supplementary Figure S4, the rarefaction curves approached a plateau, indicating that the sequencing depth was adequate for microbiome analysis. The maximum sequencing depth that allowed the retention of all samples for taxonomic analysis was approximately 100,041 sequences per sample.

#### 3.7.2 Species accumulation boxplot

The species accumulation boxplot was used to evaluate sampling adequacy across the study population. As shown in Supplementary Figure S10, the species accumulation curve increased progressively with the number of sequenced samples and approached a plateau after all 28 samples had been included, indicating that the sampling effort was sufficient to capture the majority of the oral microbial diversity present in the study population.

#### 3.7.3 α-diversity

A diversity was assessed using the Chao1, Shannon, and Simpson indices. The Shannon and Simpson indices showed only modest differences in oral microbial diversity between thalassemia patients and healthy controls.

In this study, the Shannon index values were 7.26 in the thalassemia group and 7.09 in the control group. Simpson index values were similar between the two groups (0.96 vs. 0.96). The Chao1 index values were 2700.784 for thalassemia group and 2414.509 for the control group, suggesting slightly higher species richness in thalassemia patients (Supplementary Figure S6 and Supplementary Table S1).

#### 3.7.4 β-diversity analysis

**FIGURE 6:**
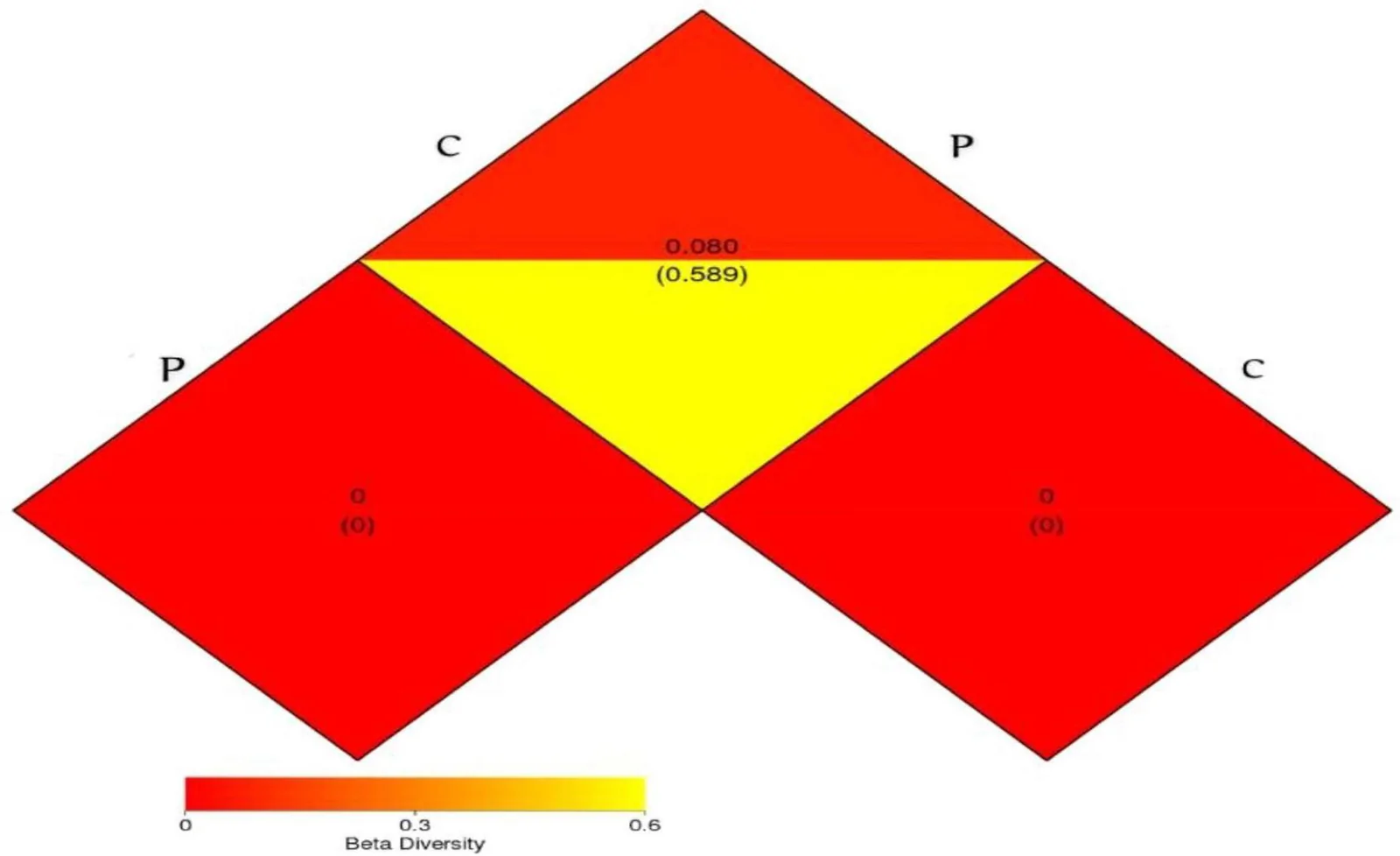
Heatmap of weighted and unweighted Unifrac β diversity measures of oral microbiomes collected from thalassemia patients (P) and healthy individuals (C).

Principal coordinate analysis (PCoA) based on weighted and unweighted UniFrac distances was used to evaluate differences in microbial community composition between thalassemia patents and healthy controls. The PCoA plots showed partial clustering between the study groups (Supplementary Figure S8). Heatmap analysis demonstrated a moderate β-diversity distance in weighted UniFrac (0.080) and a higher β-diversity distance in unweighted UniFrac (0.589) (Figure 6 and Supplementary Figure S7). These findings suggest that differences between the study groups were more apparent in the presence or absence of microbial taxa than in their relative abundances.

## 4 Discussion

In this study, we characterized the oral microbiota of thalassemia patients and healthy individuals from the Saudi Arabian population using 16S rRNA gene sequencing. A balanced microbiota is essential for maintaining overall wellbeing. However, pharmacological, medical, nutritional, or physiological changes in the host may alter the oral microbiota, potentially contributing to oral and systemic health disturbances. Even minor environmental changes may influence microbiome composition, gene expression, metabolic activity, inter-microbial interactions, and potentially the host’s emotional wellbeing (Tagg et al., 2023).

In this study, 16S rRNA gene sequencing data from 28 oral samples collected from thalassemia patients and healthy individuals were analyzed. Overall, only modest differences in bacterial composition, oral microbiota richness, and diversity were observed between the two groups. α-diversity metrics indicated a slight increase in microbial richness in thalassemia patients compared with healthy controls. These observations are consistent with previous studies reporting increased oral microbiota diversity in thalassemia patients (Aggarwal et al., 2023). PCoA showed partial separation between the two groups, although clear clustering was not observed. ANOSIM analysis showed considerable variability within both groups, which may reflect individual differences in lifestyle, diet, and other host-related factors that were not assessed in the present study. These findings underscore the need for larger-scale studies to confirm and expand upon these observations.

Taxonomically, nominal differences in bacterial abundance were observed between the two groups. At the genus level, differences were observed for *Prevotella* (raw *P* = 0.001), while at the species level, differences were observed for *Prevotella pallens* (*Prev. pallens*), *Prevotella sp.*, *Porphyromonas gingivalis* and *Eubacterium sulci* (raw *P* = 0.016, *P* = 0.034, *P* = 0.026, *P* = 0.018, respectively). However, statistical significance was not observed for the identified taxa after Benjamini–Hochberg FDR correction. Therefore, these findings should be considered exploratory and require confirmation in larger independent studies.

Overall, the taxa showing nominal differences between thalassemia patients and healthy individuals belonged to two phyla Firmicutes and Bacteroidota. Firmicutes was the most abundant phylum in both groups, consistent with recent studies reported elsewhere (Al-Sarraj et al., 2024). Thalassemia patients showed slightly lower relative abundances of Firmicutes and Bacteroidota, whereas Actinobacteriota and Proteobacteria were more abundant, in agreement with recent studies (Nonejuie et al., 2024). These phylum-level differences may reflect broader shifts in the oral microbiome composition.

The present study observed a lower relative abundance of Bacteroidota in thalassemia patients. Differences in the abundance of *Prevotella*, a member of the Prevotellaceae family within the Bacteroidota phylum, were also observed. A lower abundance of Prevotella in thalassemia patients has been reported in a recent study (Nonejuie et al., 2024). Differences in *Prevotella* abundance between male and female subgroups were also observed in the present study and are consistent with previous findings reported by Tett et al. (2021). Nevertheless, the subgroup analyses were based on a limited number of participants; therefore, these observations require confirmation in larger cohorts. Previous studies have associated *Prevotella* with plant-rich diets and carbohydrate fermentation (Christensen et al., 2019; Iljazovic et al., 2021; Tett et al., 2021), as well as glucose homeostasis (Byrne et al., 2015; Hjorth et al., 2018). However, because dietary and clinical metadata were not available in the present study, the factors underlying the observed differences in Prevotella abundance remain uncertain.

*Eubacterium sulci* is generally considered a low-abundance member of the oral microbiome. In the present study, nominal differences in the abundance of *E. sulci*, *Prevotella* sp., and *Prevotella pallens* were observed between thalassemia patients and healthy individuals. Higher abundance of *Prev. pallens* has previously been reported in females compared to males (Könönen and Gursoy, 2022). Similarly, it was reported elsewhere that *E. sulci, P. pallens*, and *Prevotella* sp., were more abundant in healthy individuals than in patients with asthma, suggesting that the abundance of these taxa may vary across disease conditions (Chiu et al., 2020). These microorganisms are generally regarded as members of the normal oral microbiota (Chiu et al., 2020; Könönen and Gursoy, 2022). In contrast, *P. gingivalis* showed a higher relative abundance in the thalassemia patients. Recent research by Kuziak et al. demonstrated that *P. gingivalis* possesses virulence factors capable of modulating host inflammatory responses (Kuziak et al., 2025). Although this observation may be biologically relevant, the present study was not designed to determine causal relationships, and the association requires confirmation in larger cohorts.

The present study has several limitations. First, the cohort size was relatively small, particularly for the male and female subgroup analyses, limiting the reliability of subgroup comparisons. Second, comprehensive clinical metadata, including thalassemia subtype, ferritin status, transfusion burden, dietary habits, oral hygiene practices, medication history, and socioeconomic factors, were not available within the approved ethical framework of this study. These variables may influence oral microbiome composition and could not be accounted for in the present analysis. In addition, functional analyses such as shotgun metagenomics, metabolomics, or transcriptomics were not performed. Finally, although several taxa showed nominal differences based on raw *P*-values, none remained statistically significant after Benjamini–Hochberg FDR correction, likely reflecting the limited sample size and the high dimensionality of microbiome data. Accordingly, the findings of the present study should be regarded as preliminary and hypothesis-generating until validated in larger, independent cohorts.

## 5 Conclusion

In conclusion, this study identified modest differences in the oral microbiome composition of thalassemia patients compared with healthy individuals, particularly at the genus and species levels. Nominal differences in the relative abundance of *Prevotella, Prevotella pallens, Prevotella* sp., *Eubacterium sulci*, and *Porphyromonas gingivalis* were observed. However, none of these associations remained statistically significant after Benjamini–Hochberg FDR correction. Therefore, these findings should be considered exploratory and warrant further investigation in larger, well-characterized cohorts. Future studies incorporating larger sample sizes and functional microbiome analyses, including metagenomics and metabolomics, may provide a better understanding of the relationship between the oral microbiome and thalassemia.

## Data Availability

The datasets presented in this study can be found in online repositories. The names of the repository/repositories and accession number(s) can be found at: https://www.ncbi.nlm.nih.gov/, PRJNA1478113.
