# Supplementary material for: Characterization of oral microbiome alterations in thalassemia patients using 16S rRNA gene sequencing
Source: Front Microbiol. 2026 Aug 17;17:1853312. doi: 10.3389/fmicb.2026.1853312 (PMC13527025; doi:10.3389/fmicb.2026.1853312)
Supplement: Supplementary file 1 [file Data_Sheet_1.pdf]

Supporting Data

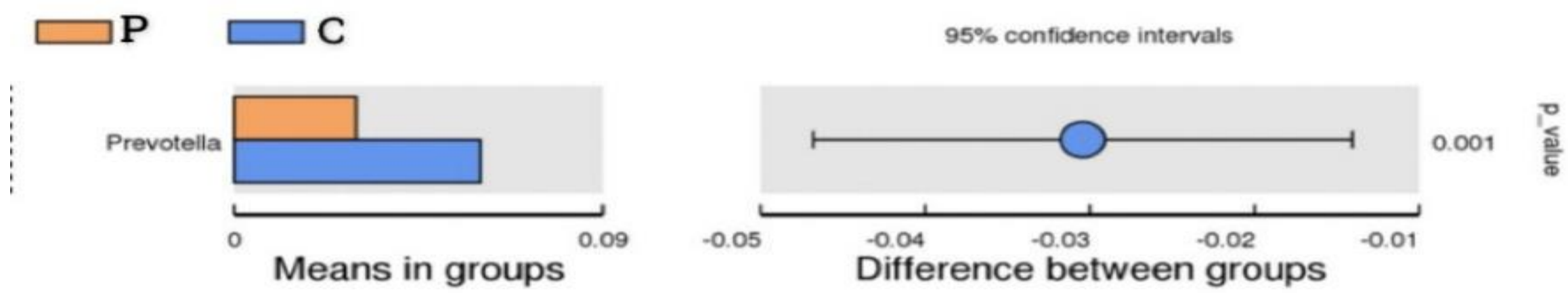

Figure S1. Bar plots displaying t-test results at the genus level for the most abundant taxa that showed significant differences between patients with thalassemia (P) and healthy individuals (C).

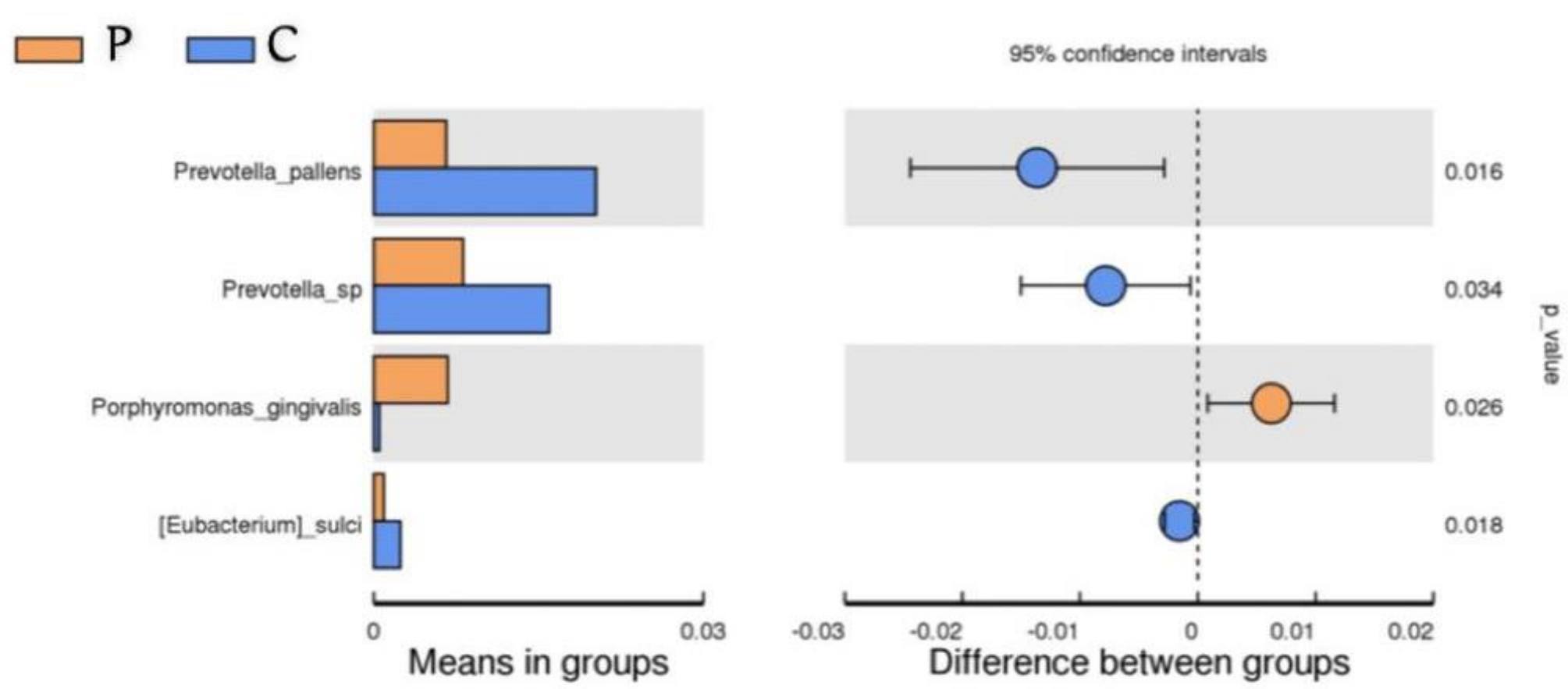

Figure S2. Bar plots displaying t-test results at the species level for the most abundant taxa that showed significant differences between patients with thalassemia (P) and healthy individuals (C).

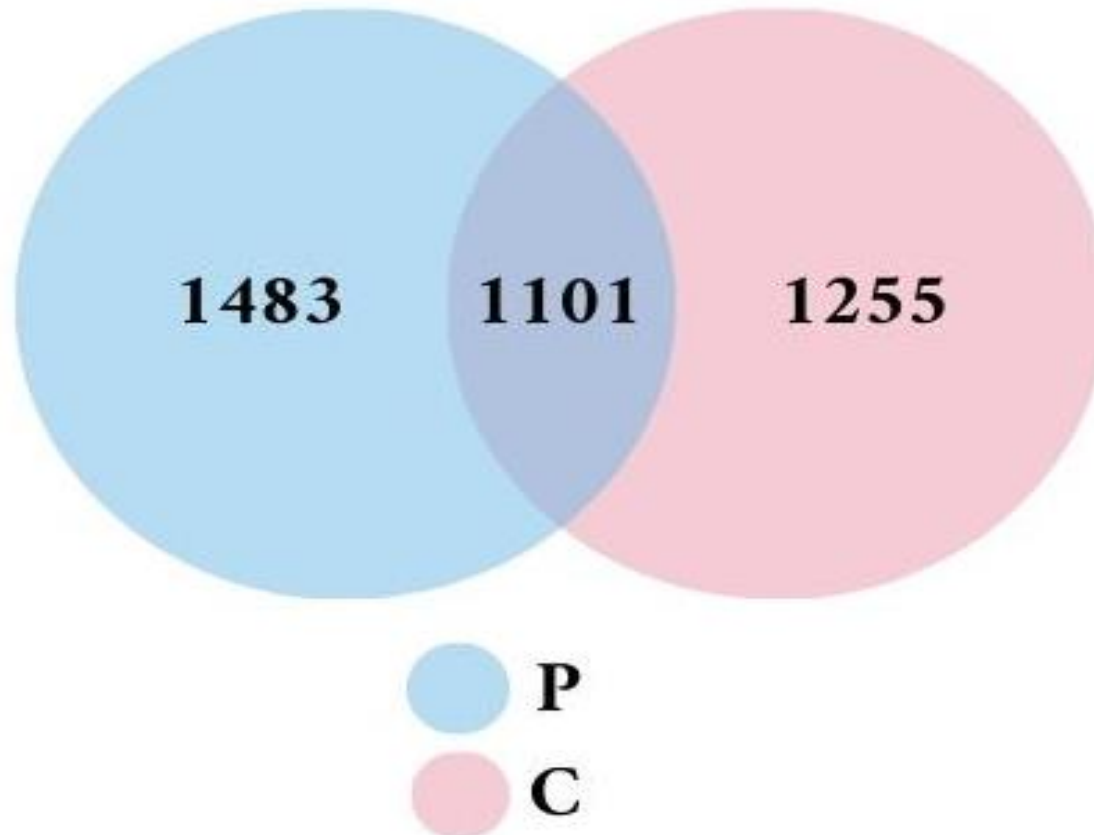

Figure S3. Venn diagram describing the unique (1483 for P and 1255 for C) and shared ASVs (1101) between the two groups of samples patients (blue) and control (pink). P = patients, C = control.

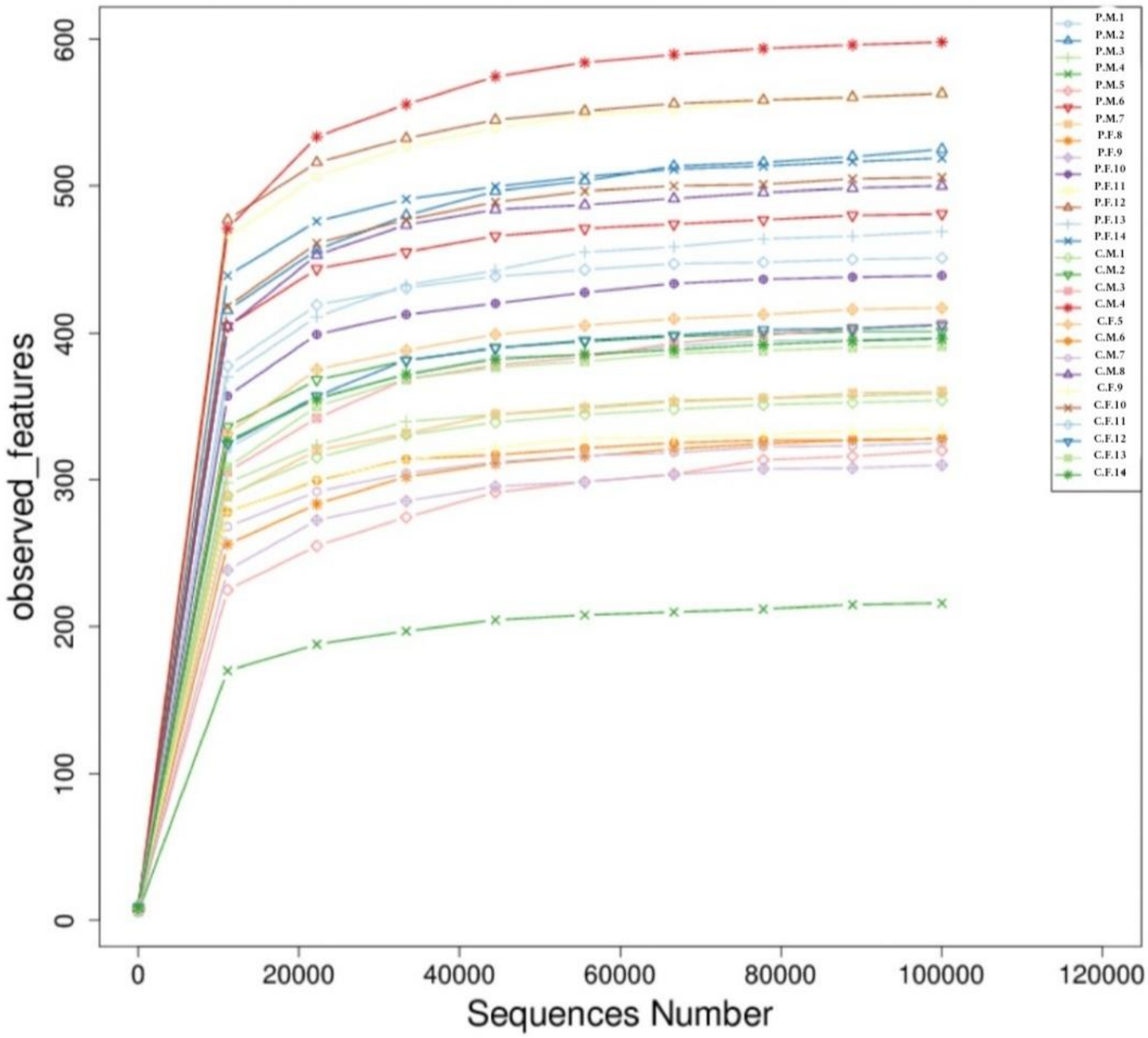

Figure S4. The rarefaction curve was generated based on the number of ASVs, representing the number of observed species (Sobs). The maximum sequencing depth used to retain samples for further diversity and taxonomic analysis was set at 100041 sequence reads.

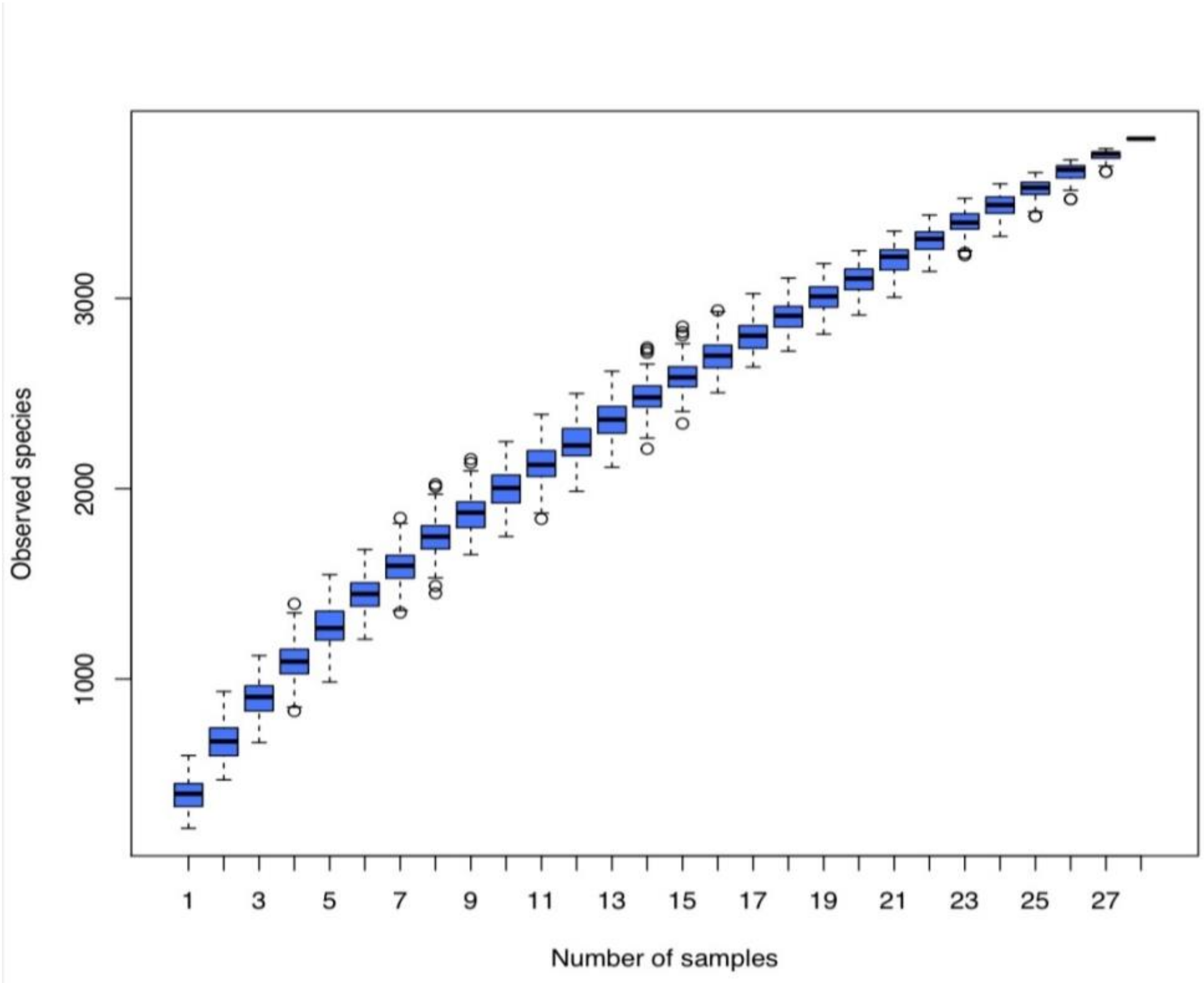

Figure S5. Specaccum or species accumulation curve of 28 oral microbiome samples collected from patients with Thal and healthy individuals.

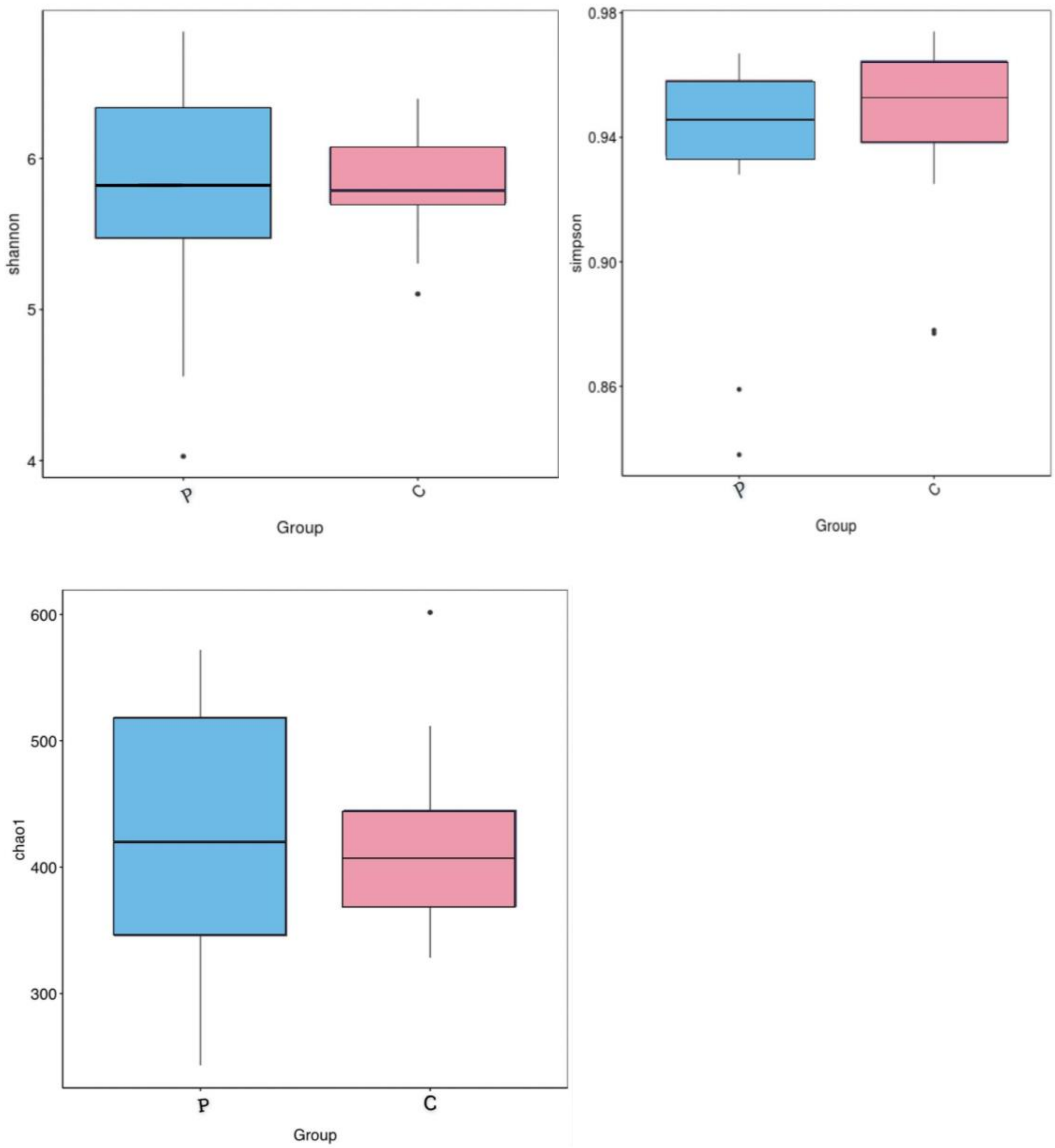

Figure S6. Alpha diversity indices as boxplots to describe richness and evenness at the group level of the patient (P) samples (blue) and control (pink).

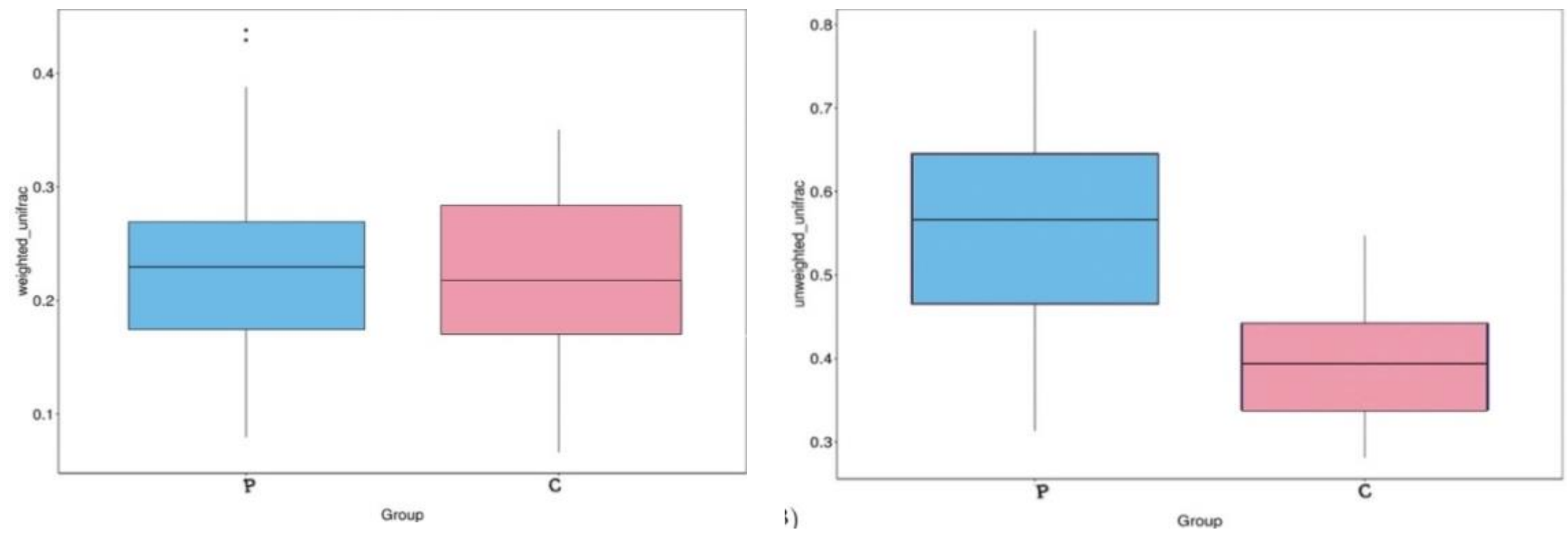

Figure S7. Boxplots show beta diversity of microbial communities based on weighted UniFrac and unweighted UniFrac distances.

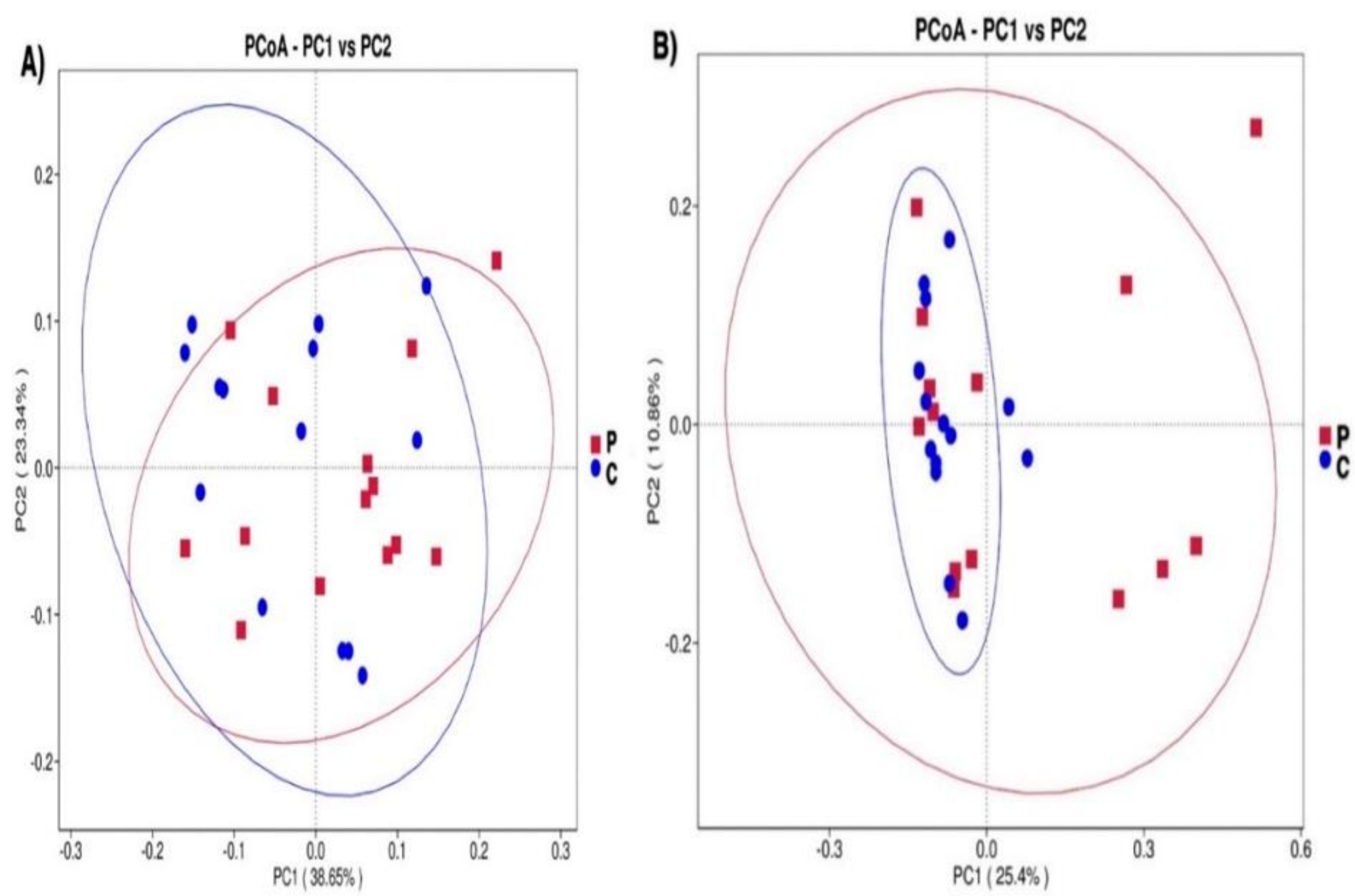

Figure S8. PCoA depends on the ASV abundance of various samples. Sample mean control is shown by a blue box. The mean patient sample is shown by a red box. The first primary coordinate is the X-axis, and the second is the Y-axis. The values in brackets show how much PCoAs contributed to the variations between samples. Every sample is represented by a dot, and various groups are represented by different colors. A)Weighted UniFrac. B)Unweighted UniFrac.

Supporting Table.

Table S1. Alpha-diversity indexes statistics among all 28 samples collected from Thal patients and the control group.

Sample_Name	chao1	shannon	simpson
**P.M.1**	400.235	5.898	0.947
**P.M.2**	546.136	6.359	0.959
**P.M.3**	365.067	5.610	0.948
**P.M.4**	243.200	4.028	0.859
**P.M.5**	339.345	5.034	0.928
**P.M.6**	484.438	6.323	0.961
**P.M.7**	367.000	5.793	0.956
**P.F.8**	330.037	5.424	0.945
**P.F.9**	316.000	4.557	0.838
**P.F.10**	442.667	5.794	0.938
**P.F.11**	572.000	6.340	0.935
**P.F.12**	571.053	6.840	0.967
**P.F.13**	474.440	5.674	0.934
**P.F.14**	529.929	6.366	0.964
**C.M.1**	360.067	5.776	0.939
**C.M.2**	401.353	6.149	0.966
**C.M.3**	422.714	5.304	0.877
**C.M.4**	601.621	6.001	0.943
**C.F.5**	420.792	5.708	0.951
**C.M.6**	328.375	5.434	0.938
**C.M.7**	332.091	5.703	0.955
**C.M.8**	507.000	6.185	0.967
**C.F.9**	337.750	6.097	0.971
**C.F.10**	511.688	5.992	0.925
**C.F.11**	452.400	6.395	0.974
**C.F.12**	408.882	5.730	0.960
**C.F.13**	395.091	5.103	0.878
**C.F.14**	403.583	5.790	0.957
